# Preconceptual Spectral and Temporal Cues as a Source of Meaning in Speech and Music

**DOI:** 10.3390/brainsci9030053

**Published:** 2019-03-01

**Authors:** Mark Reybrouck, Piotr Podlipniak

**Affiliations:** 1 Musicology Research Group, KU Leuven–University of Leuven, 3000 Leuven, Belgium; 2 IPEM–Department of Musicology, Ghent University, 9000 Ghent, Belgium; 3 Institute of Musicology, Adam Mickiewicz University in Poznań, ul. Umultowska 89D, 61-614 Poznań, Poland; podlip@amu.edu.pl

**Keywords:** preconceptual meaning, affective vocalizations, action-oriented embodied approach, affect burst, speech prosody, musical expressiveness

## Abstract

This paper explores the importance of preconceptual meaning in speech and music, stressing the role of affective vocalizations as a common ancestral instrument in communicative interactions. Speech and music are sensory rich stimuli, both at the level of production and perception, which involve different body channels, mainly the face and the voice. However, this bimodal approach has been challenged as being too restrictive. A broader conception argues for an action-oriented embodied approach that stresses the reciprocity between multisensory processing and articulatory-motor routines. There is, however, a distinction between language and music, with the latter being largely unable to function referentially. Contrary to the centrifugal tendency of language to direct the attention of the receiver away from the text or speech proper, music is centripetal in directing the listener’s attention to the auditory material itself. Sound, therefore, can be considered as the meeting point between speech and music and the question can be raised as to the shared components between the interpretation of sound in the domain of speech and music. In order to answer these questions, this paper elaborates on the following topics: (i) The relationship between speech and music with a special focus on early vocalizations in humans and non-human primates; (ii) the transition from sound to meaning in speech and music; (iii) the role of emotion and affect in early sound processing; (iv) vocalizations and nonverbal affect burst in communicative sound comprehension; and (v) the acoustic features of affective sound with a special emphasis on temporal and spectrographic cues as parts of speech prosody and musical expressiveness.

## 1. Introduction

The problem of meaning extraction in speech and music has received a lot of concern in different fields, such as infant-directed speech and singing, the origins of music perception and cognition, and the primary use of acoustic cues in emotion-driven and affect-laden preverbal communication. This kind of research saw its heyday in the 1990s with major contributions in the field of early music perception [1,2] and preference. Many efforts have been directed towards the study of *motherese* and *infant-directed speech* and *singing* [3,4,5,6,7], the acoustic basis of young children’s preference for such kinds of vocal communication [8,9], the musical elements in early affective communication between newborns and caregivers [10,11,12], and the role of prosodic features in preverbal and early musical communication [13,14,15]. Most of this research has stressed the extreme sensitivity of young infants for acoustic features of speech and music [16] as well as the existence of early musical predispositions [17,18,19]. 

Many of these studies—and also some subsequent ones—have emphasized certain commonalities between language and music [19,20,21], most of them being related to the *prosodic* and *paralinguistic features* of language, which can be considered as being musical to some extent. Besides, there are lots of empirical results which indicate that both music and language can prime the meaning of a word and that music meaning is represented in a very similar fashion to language meaning in the human brain [22,23]. This observation suggests that propositional semantics that is specific solely to language can be based on broader meaning categories. which are less precise, but not language specific. 

Recent developments have provided additional evidence from the domains of *comparative* [24,25] and *evolutionary musicology* [26,27,28,29,30,31,32,33], which deal with the evolutionary origins of music by adopting a comparative approach to vocal communication in animals and an evolutionary psychological approach to the emergence of music in the hominin line [34]. These approaches make it possible to tease apart those processes that appear to be innate from those that develop with maturation or acculturation [35]. The animal research provides insights in the role of acoustic cues in nonverbal and preverbal communication [36,37], which are related to affective speech and which can be considered emotional antecedents of music and language [15]. Some of these findings seem to corroborate, to some extent, Darwin’s hypothesis on musical protolanguage, which stated that speech and music originated from a common precursor that developed from “the imitation and modification of various natural sounds, the voices of other animals, and man’s own instinctive cries” [38] (p. 3). Such a primitive system would have been especially useful in the expression of emotion and music, as we know it nowadays, and should be a behavioral remnant of this early system of communication (see also [39,40,41]). It is a hypothesis which has been elaborated and restated by modern researchers under the umbrella of the musical protolanguage hypothesis [20,24,40,42,43].

## 2. Meaning Before Language and Music

Meaning can be considered as the result of the interpretation of stimuli by the nervous system. Such interpretation is often described in terms of internal mental representations that animals have of the things, events, and situations in their environment, and it is evolutionary older than their corresponding expressions in language [44] (pp. 4–6). From a phylogenetic perspective, there are three major kinds of meaning which have evolved over time: Meaning as a way to orient oneself in the environment [45], emotions and emotional communication as integral decision mechanisms [46] and as motivational states which are meaningful for survival by providing also a primordial way of interpretation of the external world [47], and referential meaning as the outcome of the appearance of a conceptual mind [48]. Meaning, moreover, can be considered as a basis for communication, using sound as the main source of information as is the case in primate and human vocalizations [49,50]. The latter, however, should not be identified solely with speech and singing, which can be contrasted clearly with nonverbal utterances, such as laughter, crying, and mourning. As such, there is a hierarchy in the kind of meaning that is conveyed by sound: There is, first, a distinction between the digital (speech and music) and analog (prosody, laughter, etc.) usage of sound [51]; there is, second, a hierarchical distinction between preconceptual and conceptual meaning with a first level of simple spectral and temporal cues as a source of reflexes and a second level of expressive dynamics and emotional communication [51,52]; there is, third, a level of meaning that is conveyed by means of syntax, such as, e.g., tonality in music and grammatical correctness in speech [53]; and finally, there is a fourth level of propositional semantics and associative meaning [48], such as language’s lexicons and, most probably, chimpanzees’ referential grunts [54].

Speech is closely related to vocal production and can be studied from a broader stance, including the articulatory, linguistic, and information-conveying point of view. The *articulatory approach* describes lexical units in terms of gestures that are characterizations of discrete, physical events that unfold during the speech production process [55]. They can be considered basic units of articulatory action, allowing phonology to be described as a set of relations among “phonological events” [56]. These basic units of articulatory routines are discrete gestures, which emerge pre-linguistically as early and gross versions of their adult use [55], calling forth the *linguistic level* of speech processing with articulatory routines that gradually develop into higher-level phonological units that can be contrasted with each other [57]. Linguistic meaning, however, is discrete-digital rather than analog-continuous. It relies on propositional knowledge without direct coupling with the speech signals—as sounding and thus sensory phenomena—and combines referential meaning with particular sound patterns that function as vehicles to convey symbolic meaning. Such a “vehicle mode” of meaning involves referential meaning, which is a representational mode of conveying information, as against the “acoustic mode”, which refers merely to the local modulations in sound that are involved in expressive communication of meanings [58].

Speech, as opposed to language as a system, is articulated in real time. As such, it is a sensory rich stimulus. It provides information across multiple modalities, combining both the auditory and visual modalities, as exemplified most typically in the facial expression of audio-visual emotional speech. The latter, together with prosody, cannot be reduced to the control of voice qualities alone, but is closely related to the integration of sensory modalities—with facial and vocal expressions reinforcing each other [13]—and even with the movements of body [59]. Much research on emotional speech (see e.g., [60]), however, has been oriented rather narrowly to facial expressions since it has been hypothesized over a long period of time that judges are more accurate in inferring distinct emotions from facial expressions than from vocal ones. Acoustic cues, on the other hand, have been considered merely as additional features to facial expression, marking only levels of physiological arousal that are less distinctive than those expressed by the face. This conclusion, however, has proved to be erroneous since previous studies have studied only a limited number of acoustic cues, and the arousal differences within emotion families have also been largely neglected [61]. This has been shown in recent studies that used a comprehensive path model of vocal emotion communication, encompassing encoding, transmission, and decoding processes [62,63] to empirically model data sets on emotional expression and recognition from two different cultures and languages. Results of their extended Brunswikian “lens model” [64]—lens equations, hierarchical regression, and multivariate path analysis—, all reflect the strong evidence from past work on the role of arousal in affective communication that vocal sounds primarily convey the arousal state of the sender. It was stated that the “voice is the privileged modality for the expression and communication of arousal and activation, whereas the face is vastly superior with respect to valence” [62] (p. 24). 

Additional evidence comes from studies of infants’ reactions to parental communicative signals, which have stressed their outstanding discriminative abilities for timing patterns, pitch, loudness, harmonic interval, and voice quality [65]. It seems, moreover, that newborns are very sensitive also to facial expressions, vocalizations, and hand movements, which they can largely imitate to some extent. Such a kind of *communicative musicality*, as it has been coined [11,66], shows children’s awareness of human communicative signals. It is a faculty which is comprehensive, multimodal, and coherent at birth and in the first months after birth [67]. It stresses the conflation of perceptual and motor aspects in speech recognition and vocal expression, bringing together audio-visual, visual-motor, and audio-motor integration. 

Music is related to this preverbal communicative expressivity. It precedes or bypasses verbal communication by stressing the sensory richness of the stimuli. As such, it is directed primarily to itself with meaning being self-referential rather than referring to something else. Contrary to the centrifugal tendency of linguistic meaning, where the attention is directed away from the text proper (centrifugal) to grasp the meaning of what is referred to, music has a centripetal tendency in directing the listener’s attention to the auditory material of the sounding music itself [68,69]. As such, there seems to be a major distinction between language and music, though there are also some commonalities, which stress a number of shared components. This applies in particular to vocal music and its communicative possibilities.

Music, seen from an evolutionarily point of view, is one of the most ancient forms of human communication, with the human voice being probably the most ancestral instrument in human music [70]. It can even be questioned in this regard whether music and speech are different, and if so, to what extent [71]. There are two ways to address this question, either by intraspecies or interspecies comparison. An example of the former is the study of para-musical elements in language and para-lingual elements in music [72], such as the use of lexical tone in tone languages and prosody (para-musical) or the use of Leitmotive in music (para-lingual) [20]. Also, the languages based on musical tone systems, such as drum and whistle languages, can be studied in this context [73]. The interspecies comparison, on the other hand, is still more challenging and embraces a very extensive body of research. It has been hypothesized, for example, that singing could have evolved from loud calls by nonhuman primates, such as the Old-World monkeys and apes, which have been considered to be possible precursors of human singing and music. Gibbons, in particular, use vocalizations that elicit emotional responses from human listeners by using acoustic characteristics, such as loudness, acceleration of note rhythm, a final slow-down in rhythm, sounds consisting of alternated exhalation and inhalation, higher pitch frequencies in the central section of the call, pure tone of notes, and frequent accompaniment with piloerection and locomotor displays [36]. All these elements, however, are also used to a different degree in speech.

As such, there is an ability of communication by means of sounds that touches on an evolutionarily old layer of sound communication, which is older than singing and speech. This level is involved in the development of functional sensitivity to a specific class of sounds in ancestral vertebrates both as an aid in identifying and localizing predators and for capturing prey [74]. It is exemplified most typically in the use of alarm calls, which can be considered as a class of punctuate sounds that tend to be short, with sharp and abrupt signal onset, dramatic frequency and amplitude fluctuations, and a chaotic broadband spectral content. There is also a broad class of vocalizations that has been labeled “squeaks, shrieks, and screams” and which have direct impact on animal perception [75]. Their specific designs make them stand out against background noise so as to make them easy to localize. Moreover, they may provoke immediate orienting reactions by other animals in the direction of the calls, in combination with reflexive movements that prepare for flight [76]. Such generalized startle responses are induced also in very young infants, even in the absence of significant previous experience. They are, in fact, reducible to the operation of low-level brainstem and subcortical processes, which are associated with sound localization, orienting, and autonomic responding [77,78]. These vocalizations, however, can be exemplary of an intentional, communicative use of sounds which differ functionally from simple auditory sensations, which are prelinguistic default labels of sound sources [79], such as sensation of loudness and low pitch, as a tag of a big animal.

Such vocalizations by animals are not gratuitous. They are used frequently by youngsters as an opportunity to influence the behavior of older and larger individuals by engaging their attention, arousal, and concomitant behavior, sometimes in a very compelling way [80]. It can be questioned, however, whether primates have a theory of mind or act intentionally to influence others. A tentative answer can be found in comparable research in humans into to the neurocognitive mechanisms (auditory prosodic activations) that allow listeners to read the intentions of speakers from vocal prosodic patterns, and which illustrates their anchoring at the interface between auditory and social cognition, involving the cooperation of distributed auditory prosodic, sociocognitive, and cingulo-opercular brain areas [81]. 

These attention-capturing sounds in animals are often characterized by loud protracted bouts of harsh and variable vocalizations, which include rapidly varying combinations of loud, noisy screams and piercing high-frequency tonal cries, with dramatic amplitude and frequency modulations, which together are able to increase the arousal state of the mother, including human ones [74,82]. It has been shown, moreover, that screaming is one of the most relevant communication signals in humans for survival. By using a recently developed, neurally informed characterization of sounds (modulation power spectrum) see [83,84], it has been demonstrated that human screams cluster within a rather restricted portion of the acoustic space between about 30 and 150 Hz, which corresponds to the perceptual attribute of roughness. This acoustic roughness has been found also to engage subcortical structures, which are critical to the rapid appraisal of danger [85].

The vocal repertoire of most primate species, however, is not limited to these attention-capturing sounds. There is also an additional class of sounds, which are referred to as “sonants and gruffs” and which may be considered as structural opposites of these arousal-increasing sounds [74]. Instead of being unpatterned and chaotic, they are tonal and harmonically rich, with a more diffuse regularly patterned broadband spectral structure. Rather than having direct impact on listener’s arousal and affect, they seem to induce a less inherent affective force. Their richly structured spectra, moreover, make them even suited for revealing clear cues to the caller’s identity since their individual idiosyncrasies impart individually distinctive voice cues that are associated either with the dynamic action of the vocal folds or with the resonance properties of the vocal tract cavities [86,87]. Chimpanzees, likewise, are able to intentionally use grunts as referential calls and to learn new calls from other individuals [54], which represents most probably an early stage of the evolution of lexical meaning (but see [88]). However, although the monkeys’ vocal tract is ready to generate speech sounds [89], language and music seem to necessitate more elaborate neural processing mechanisms and vocal control [46]. 

## 3. Affective Sounds and Vocalizations

Speech—at least in its most primitive appearance—and music seem to share a common affective substrate. Studying emotional communication by means of speech and music, therefore, can benefit from a thorough investigation of their underlying mechanisms. One field of research that has been particularly fruitful in this regard has been the study of auditory affective processing that was conducted in the context of *speech prosody* [13]. It has been argued, in fact, that two separate neuroanatomic channels with different phylogenetic histories participate in human acoustic communication to support either nonverbal affective vocalization or articulate speech [90,91]. This *dual-pathway model* of human acoustic communication clearly distinguishes the propositional and emotional contents of spoken language, which rely on channels that are seated in separate brain networks that create different data structures, which are known as analogue versus digital (see below). Both channels, however, must coordinate to some extent, but the functional mechanisms and neuroanatomic pathways underlying their intertwined integration are still not totally clear [92].

Affective prosody, further, is opposed to the discrete coding of speech, which is used in the case of phonemes, words, and those aspects of music that consist of pitches and durations. Its expressive dynamics can be modelled more effectively by continuous variables, as is the case with emotional gestures that are shared not only by all humans, but also by a broader group of animals, including many taxa of mammals and even other vertebrates [51]. The same dynamics of affective prosody—as an evolutionarily old form of communication—are to be found, in fact, in the prosody of human language and in the vocal expressions of different mammalian species, which could mean that its use in human acoustic communication has deep phylogenetic roots that are present in the vocal communication systems of nonhuman animals as well. Consistent structures, in fact, can be seen in acoustic signals that communicate affective states, such as high-pitched, tonal sounds in expressions of submission and fear, and low, loud, broadband sounds in expressions of threats and aggression. Animal signals may thus have direct effects on listeners. They may not simply provide information about the caller, but may effectively manage or manipulate the behavior of listeners [93] (see also [76]). This *prehuman origin hypothesis* of affective prosody locates its grounding in innate mechanisms, which have a prehuman basis and which are used to discriminate between different emotions, both qualitatively (anger, fear, joy, sadness, boredom, etc.) and quantitatively (affect intensity) [52]. It has been shown, moreover, that there exists a functional dissociation between brain regions that process the quality of acoustically conveyed emotions (orbitofrontal cortex) and those that process the intensity of that emotion (amygdala) [94]. Current research has also revealed a high degree of acoustic flexibility in attention-attracting sounds in nonhuman mammalian species, which points in the direction of more complex acoustic signaling and processing mechanisms [95].

As such, it can be argued that the study of the faculties of language and music can benefit from a comparative approach that includes communication and cognition in humans and nonhuman animals alike [46]. The capacity to learn language, in fact, requires multiple, separable mechanisms, which include the ability to produce, perceive, and learn complex signals as well as to interpret and control them. Some of them seem to have figured already in the common ancestors of both humans and animals, some others evolved later. Relying on comparative data from living animals, therefore, may be definitively helpful to address these issues. Acoustic signaling in humans, in this view, may have roots in the vocal production, auditory perception, and cognitive processing capabilities of nonhuman mammals, and the study of affective prosody, as a shared component of human speech, music, and nonverbal acoustic communication, in particular, may shed some light on the evolutionary roots of human speech and music as well as the evolution of meaning itself. It is important, in this regard, to consider also the role of *iconicity*—the similarity between some aspects of sound to some aspects of meaning—in linking the sound to meaning in language. It should be noted, in fact, that affective prosody is considered a paralinguistic property, which accompanies the semantic meaning arising from the symbolic system of human language. The question of how meaning emerges from symbolic signs, therefore, cannot be fully understood by focusing only on prosodical features of language, which work in parallel to the semantic processing. Here, an iconic relationship between sound and the meaning of words that has traditionally been considered as only a marginal property of language (e.g., onomatopoeia, and to some extent also phonaesthemes, i.e., a phoneme or group of phonemes, which has recognizable semantic associations as the result of appearing in a number of words with similar meanings, such as, e.g., the English onset /sn-/ in snarl, snout, sniff, snuffle), has been assumed to serve as an interface for accomplishing the need to map linguistic form to human experience as a vital part of meaning making. Iconicity, thus, has been shown to play an important role for both phylogenetic language evolution (e.g., [96]) and ontogenetic language development (e.g., [97]). This holds in particular for the correspondences between the sound and meaning of words in the affective domain, termed *affective iconicity* [98], which have been supported by recent empirical results indicating that the specific sound profile of a word can be attributed to a specific affective state, which, in turn, can contribute to the perception of the affective meaning of that word, such as, e.g., whether it designates something positive/negative or arousing/calming [99]. Importantly, the affectivity in the sound of words in a language has been shown to be processed in similar brain regions that are involved in processing other types of affective sounds, such as emotional vocalization and affective prosody [100,101]. In addition, such affective potential in the sound of words is even capable of interacting with higher cognitive processes, such as affective evaluation of the words’ meaning [102]. All this suggests that consciously experienced meaning is inferred from a number of cues that reflects a hierarchy of sound processing.

It is possible, further, to conceive of this hierarchy in the processing of sounds, reflecting the evolutionary history of human sound communication from early mammals, showing an extension of the perceivable spectrum of sound frequency related to the evolution of the mammalian ear [103], to primates. Non-human primates and early hominins, for example, are an especially interesting group in which to consider the potential affective influence of vocalizations on listeners. Because of their large brains and their phylogenetic proximity to humans, traditional research has focused mostly on “higher-level” cognitive processes that organize communication in higher primates. Yet, they still can rely on the neurophysiological substrates for affective influence, which are still very broadly conserved. It is likely, therefore, that affective influence is an important part of the vocal signals of non-human primates [74]. As such, it is possible to conceive of hierarchical levels of affective signaling, starting from loud calls and vocalizations of early hominids, over prelinguistic affective processing of sound by neonates to infant-directed speech, affective speech, and even music. The step via onomatopoeia and iconicity, finally, could be added as a last step from affective to referential signaling.

The loud calls of *early hominins* are exemplified most typically in a broad class of vocalizations with acoustic features that have direct impact on animal perception, as mentioned already above: Sharp signal onsets, dramatic frequency and amplitude fluctuations, and chaotic spectral structures [104]. *Neonates* are another interesting group for the study of prelinguistic affective processing of sound. They have been shown to possess complex endowments for perceiving and stimulating parental communicative signals by discriminating timing patterns, pitch, loudness, harmonic interval, and voice quality [65]. They also seem to react to the human voice and display imitations of facial expressions, vocalizations, and hand movements, showing an awareness of human signals that is already comprehensive, multimodal, and coherent at birth [67]. As a result, people, all over the world, have capitalized on this sensitivity by developing *infant-directed speech* or *motherese* (see below), which is obviously more simplified than adult speech, and which involves exaggerated prosodic features, such as wider excursions of voice pitch, more variable amplitude, tempo, and delivery, and more varied patterns of word stress [74]. All these features have been the subject of research on auditory affective processing, which has been conducted mainly in the context of speech prosody, which has been coined also the “third element of language” [105]. Vocal emotion perception in speech, further, has been studied by using test materials consisting of speech, spoken with various emotional tones by actors, and nonverbal interjections or *affect bursts*, such as laughter or screams of fear [106] (see for an overview). These vocal expressions, which usually accompany intense emotional feelings, along with the corresponding facial expressions, are closely related to *animal affect vocalizations* [107], which can be defined as short, emotional non-speech expressions, which comprise both clear non-speech sounds (e.g., laughter) and interjections with a phonemic structure (e.g., ‘Wow’), but which exclude verbal interjections that can occur as a different part of speech (like ‘Heaven’, ‘No’, etc.)” [108].

These nonverbal affect bursts have proven to be useful for the study of meaning. They provide an interesting class of affective sounds, which have been collected in validated sets of auditory stimuli—such as the Montreal Affective Voices (MAV) [106] and the “Musical Emotional Burst (MEB) for musical equivalents [109]. Using nonverbal sounds, moreover, presents several advantages over verbal ones: The stimuli do not contain semantic information, there are no linguistic barriers, the expression of emotion is more primitive and closer to the affect expressions of animals or human babies, and they are more similar to the Ekman faces [110] used in the visual modality than emotional speech. As such, they avoid possible interactions between affective and semantic content, they can be used for the study of cross-cultural differences, and they allow better comparisons across modalities, as well as studies of cross-modal emotional integration [106].

Affect bursts, however, are limited in their semantic content, but are able to communicate by sound [51,111]. Being evolutionarily older than singing and speech, they have been considered as their precursors to some extent. Singing is one of the interesting ways of sound expression, which goes beyond the transmission of semantic information. It can be questioned, however, whether every kind of music—as an evolved and cultural product—exploits such pre-existing perceptual sensitivities, which were originally evolved thanks to a variety of auditory functions, such as navigating sonic environments and communication by means of singing. Cultural evolution, in this regard, has led to increasingly complex and cumulative musical developments through processes of sensory exploitation [112].

## 4. Calls, Vocalizations, and Human Music: Affectively-Based Sound–Meaning Relationships

Music has inductive power. It can move listeners emotionally and physically by means of the information-processing mechanisms it engages. The majority of these mechanisms, however, did not evolve as music-specific traits. Some of them are related to the processing of sound that is recognized as being similar to voices, objects that are approaching, or the sounds of animals. As such, this processing seems to involve cognitive processes of attraction and cultural transmission mechanisms that have cumulatively and adaptively shaped an enormous variety of signals for social relationships [112]. Music, in this view, is an inherently social phenomenon, and the same holds true for loud calls of nonhuman primates, especially those of the Old-World monkeys, which, most likely, were the substrate from which singing could evolve [36].

This brings us to the question of the origins of language and music and their mutual relationship. It has been hypothesized, e.g., that language seems to be more related to logic and the human mind, whereas music should be grounded in emotion and the human body [113] (see for an overview). This dichotomous approach has been questioned, however, in the sense that language and music could evolve from common roots, a common musical protolanguage [24,42]. Especially, the *loud calls* in modern apes and music in modern humans seem to be derived from such a common ancestral form. The calls are believed to serve a variety of functions, such as territorial advertisement, inter-group intimidation and spacing, announcing the precise locality of specific individuals, food sources, or danger, and strengthening intra-group cohesion. The most likely function of early hominin music, on the other hand, was to display and reinforce the unity of a social group toward other groups [36]. This is obvious in vocalizing and gesturing together in time, where the ability to act musically underlies and supports human companionship. It seems likely, moreover, that the elements of communicative musicality are necessary for joint human expressiveness to arise and that they underlie all human communication [11,66].

As such, it seems that a major ancestral function of calls, protolanguage, and music may be related to several kinds of signaling, attention capturing, affective influence, and group cohesion rather than conveying propositional knowledge that is related to higher level cognitive processes that are involved in the communication of contemporary humans. This brings us to the role of *affective semantics*, as the domain that studies semantic constructs that are grounded in the perceptual-affective impacts of sound structure [74]. Empirical grounding for that kind of signaling has been provided by a typical class of primate vocalizations, which are known as *referential emotive vocalizations* [58] and separation calls [114]. There are, in fact, a number of important affective effects of sounds and vocalizations, such as, e.g., attention capturing mechanisms, which are used also in speech directed to young infants with the function to focus and maintain attention and to modulate arousal by using dramatic frequency variations. As such, there is a whole domain of acoustic signals which goes beyond the lexico-semantic level of communication and which is shared between humans and non-human animals. There are, as such, acoustic attributes of aroused vocalizations which are shared across many mammalian species and which humans can use also to infer emotional content. Humans, as a rule, use multiple acoustic parameters to infer relative arousal in vocalizations, but they mainly rely on the fundamental frequency and spectral centre of gravity to identify higher arousal vocalizations across animal species, thus suggesting the existence of fundamental mechanisms of vocal expressions that are shared among vertebrates, and which could represent a homologous signaling system [115].

Such core affective effects of vocal signals may be functional. Yet they do not undercut the role of cognition and the possibility of more complex communicative processes and outcomes, such as speech communication in people. The latter can be seen as a refinement of phylogenetically older vocal production and perception abilities that are shared with non-human animals [91]. These abilities may scaffold, in part, an increasing communicative complexity, which means that at least some of the semantic complexity of human language might capitalize on affectively-based sound–meaning relationships. It is probable, therefore, that evolutionarily older ways of interpreting acoustical cues can be involved in the construction of more complex meaning. Such preprepared or early acquired sound–sense relationships represent a form of intrinsic or original meaning that provides a natural foundation from which increasingly complex semantic systems may be constructed, both developmentally and evolutionarily. This foundation can explain the universal tendency first observed by Köhler [116] (pp. 224-225) to associate pseudowords, such as *takete* or *kiki*, with spiky shapes whereas *malumba* or *bouba* are associated with round shapes [117]. It has been shown, moreover, that the communicative importance of the affective influence of vocal signals does not disappear when brains get larger and their potential for cognitive, evaluative control of behavior increases. It is likely, therefore, that complex communicative processes exploit and build on the phylogenetically-ancient and widespread affective effects of vocal signals [74] (p. 183).

## 5. Sound Communication, Emotion, and Affective Speech

Sounds can have a considerable affective effect on listeners and this holds true also for non-human animals that use many of their vocal signals precisely to exert these effects. There is, as such, a relationship between the acoustic structure in animal signals and the communicative purposes they purport [74,112]. This is obvious in vocalizations of non-human primates, which bear the mark of design for direct effects on the listener’s affect and behavior, as exemplified most typically in alarm vocalizations that are produced during encounters with predators [91]. These alarm calls tend to be short, broadband calls, with an abrupt-onset, standing out against background noise, thus being easy to localize. As such, they display acoustic features for capturing and manipulating the attention and arousal in listeners. They have been studied already in the 1970s in the context of agonistic vocalizations that are involved in confrontations or competitions with others. Among their most important features is a low fundamental frequency (F_0_) and a tendency towards aperiodicity, with a possible explanation that low, broadband sounds with a wide frequency range are often tied to body size and hostile intent. Such sounds, presumably, can induce fear in the receivers. High pitched sounds with tone-like high F_0_, on the contrary, are related to appeasement and are often produced to reduce fear in listeners [118,119]. This illustrates again how sound is often more important than semantic meaning in animals’ signals.

Similar findings have been reported also for humans. Prohibitive utterances across cultures, for example, contain similar acoustic features, such as a fast rising amplitude, lowered pitch, and small repertoires [112]. A more elaborated field of research, however, is the study of *motherese* or *infant-directed speech* [65]. Mothers, as a rule, speak in short bursts and talk in an inviting sing-song manner with the baby occasionally answering back. Young infants, moreover, stimulate their caregivers to a kind of musical or poetic speech, which can move into wordless song with imitative, rhythmic, and repetitive nonsense sounds. Such baby–mother interactions imply communicative interactions, which have also been called “communicative musicality” [11]. They suggest an awareness of human signals which is present at birth, with newborns reacting to the human voice and imitating facial expressions, vocalizations, and hand movements. It means that young infants possess complex endowments for perceiving and stimulating parental communicative signals by discriminating timing patterns, pitch, loudness, harmonic interval, and voice quality [65]. Effective communication, in this view, must be held by means other than lexical meaning, grammar, and syntax, with mothers and babies being highly "attuned" to the vocal and physical gestures of the mother. Both seem to explore pitch-space in a methodical manner over short and long intervals of time [11]. This has been reported extensively by the Papoušeks [6,19], who both have stressed the importance of early childhood musical behaviors as forms of play to nurture children’s exploratory competence. They have studied intensively infant–caregiver interactions and focused on the musicality of these interactions, stressing the indivisibility of music and movement. It has been found, in fact, that music and movement share a dynamic structure that supports universal expressions of emotion as exemplified in particular in infants’ predispositions for perceptual correspondences between music and movement. This ability, further, seems to be possible by the existence of prototypical emotion-specific dynamic contours, but also by isomorphic structural relationships between music and movement [120].

They found out that the parent’s multimodal stimulation is, so to say, tailored to the infant’s early competence for perceiving information through different senses and that “regular synchronization of vocal and kinaesthetic patterns provides the infant with multimodal sensory information including tactile, kinaesthetic and visual information.” [6] (p. 100). Similar findings have been reported by Trevarthen [121], who has centered on the temporal characteristics of the infant–caregiver interaction. The rhythmicity of this interaction can be described as the capacity of the infant to follow and respond to temporal regularities in vocalization and movement, and to initiate temporally regular sets of vocalizations and movements. What he proposes is a conceptual framework to explore the expression and development of communication or intersubjectivity through empirical observations and analyses of infant–caregiver interaction. It enables the sharing of patterned time with others and facilitates harmonizing the affective state and interaction [27]. 

As such, there seems to be an evolutionarily old layer of sound communication that exists in speech, but that arouses emotion in singing as well. This happens in a hierarchic order with the evolutionarily older elements being most basic and effective, and those which are acquired in processes of socialization being most subtle and conventional. Primitive affective vocalizations, therefore, are considered as more authentic and more truly felt information than conventional and ritual information [10,122], and a great deal of music is also designed specifically to give rise to these affective effects [74].

## 6. Sound/Speech Understanding and the Gestural Approach

Language and music can be considered as sound-signal using communication systems. There is, however, a distinction with respect to their respective semantics, which can be either lexico-semantic or action-oriented. In language, as well as in music, the vocal or acoustic characteristics may help to convey an impression, but it has been shown that the position of the eyebrows and the facial expression as a whole, may have the same function [119]. Many facial gestures, in fact, are part of a multi-modal array of signals, and facial expressions may even influence the acoustic cues of the expression by vocal tract deformation [13].

This brings us to the question of bimodality and audiovisual integration of emotional expressions [123]. Even in visible emotion, for example, the auditory modality can carry strong information, which is not only related to the consequences of the facial gestures [13]. In this context, it is important to remind the musicality of infant–caregiver interactions with synchronous stimulation that provides continuous multimodal sensory information (see above). This multimodal stimulation, further, entails processes of affective and behavioral resonance in the sense that the neurophysiological organization of behavior depends on a reciprocal influence between systems that guides both the production, perception, interpretation, and response to the behavior of others, somewhat reminiscent of the discovery of mirror and canonical neuron systems in primate brains [124]. This means that seeing an object or an action performed by someone else can activate the same neurons as when one is performing this action oneself. However, the multimodal stimulation can be even stronger. It has been shown, for example, that if acoustic speech is the main medium for phonetic decoding, some integration with the visual modus cannot be avoided [125]. As such, there is a lot of interest in the role of the co-occurrence of sight and sound, with a special focus on research on emotion effects on voice and speech [61].

Multimodal stimulation entails interactions between individuals, which is obvious in the ability to vocalize and gesture together—as in synchronous chorusing and gesturing—both in humans and nonhuman primates [126]. The ability to act musically and to move sympathetically with each other, accordingly, seems to be the vehicle for carrying emotions from one to someone else. It underlies human companionship in the sense that elements of communicative musicality are necessary for joint human expressiveness to arise [11].

Speech, as a later evolutionarily development, pays tribute to this interactive, gestural approach. It is a basic claim of articulatory phonology, which states that articulatory gestures and gestural organization can be used to capture both categorical and gradient information [55]. They can be described as events that unfold during speech production and whose consequences can be observed in the movements of the speech articulators. Gestures, in this view, are dynamic articulatory structures, which consist of the formation and release of constrictions in the vocal tract. As such, they can be described in terms of task-dynamics, which have been used to model different kinds of coordinated multi-articulator actions, such as reaching and speaking. It means also that the same gestural structures may simultaneously characterize phonological properties of the utterance (contrastive units and syntagmatic organization) and physical properties.

## 7. Sound Comprehension in Speech and Music: Spectral and Temporal Cues

Articulatory gestures are situated at the productive level of vocal communication. There is, however, also the receptive level, which is related to the recognition of acoustic parameters, such as, for example, spectral cues when we discriminate pitch in music [127] and intonation patterns in speech [128]. Sound comprehension, in this view, should be related to the recognition of the acoustic profiles of vocal expression, as exemplified most typically in emotional expression. It has been stated erroneously that the voice might only reflect arousal. Recent research, using a larger number of parameters, has shown that spectro-temporal parameters play a major role in differentiating qualitative differences between emotions [129]. This is obvious, for example, in the vocal repertoire of most primate species with a clear distinction between squeaks, shrieks, and screams, with direct impact on the listener’s arousal and affect, and sonants and gruffs, with structured spectra that provide an excellent medium for revealing clear cues to the identity of the caller (see above). These cues, which are highly idiosyncratic, impart distinctive voice cues in the acoustic features of these calls, which are associated with the patterns of dynamic action of the vocal folds or with the resonance properties of the vocal tract cavities [74,87]. Human infants, accordingly show an impressive acoustic sensitivity, which allow them to discriminate timing patterns, pitch, loudness, harmonic interval, and voice quality [11], with many perceptual biases being in place before articulated speech evolved [112]. Importantly, although all these features depend on acoustic parameters, they are in fact auditory phenomena [79]. It means that the discrimination of vocal cues is the interpretation of sound stimuli by the nervous system influenced by genetic (both species specific and shared with other taxa) and environmental (including cultural) factors.

Music as well as speech can be considered as dynamic stimuli with sounds changing continuously across the time of presentation. This means that new sensory information is added serially during sound presentation, with physiological systems that respond to simple changes in the physical stimulus being continuously active. Sounds, moreover, are dynamic and often require an accrual of information over time to be interpreted [130]. The effects of speech and music, therefore, are related in important ways to the information-processing mechanisms they engage. As a result, humans interpret speech and music sounds not only as expressive information, but also as coherent sound structures, which convey the whole pack of information. Even at this level, however, both speech and music structures are auditory phenomena which rely to a different degree on acoustical cues. In the case of phonemes recognition [131] and timbre discrimination in music [132], the most important cues are spectro-temporal. Spectral cues, in contrast, are crucial in the discrimination of intonation patterns in speech and pitch class structure in music [127].

The main difference between speech and music in this regard consists in the role of particular acoustic cues played in the transmission of meaning. While spectro-temporal cues are crucial for the recognition of words, they seem to be less important as far as the music structure is concerned. It means that spectro-temporal cues evolved in humans as a main source of transmitting lexical meaning. In contrast, spectral cues are important for discrete pitch class discrimination in music—one of the main elements of musical structure—which is deprived of lexical meaning. Nonetheless, spectral cues can contribute to the lexical meaning in tone languages where the relative change of pitch influences the interpretation of the word meaning [133]. Even in tone languages, however, lexical meaning is conveyed mainly by the means of spectro-temporal cues. Similarly, temporal cues can be used as an additional source of information which influences lexical meaning in “quantity languages”, which are sensitive to the duration of the segments for the assignment of their meaning [134,135]. It has been shown also that spectral and temporal cues contribute to the signaling of the word meaning in non-tonal languages as well [136], with the extent to which these cues are important for the transmission of lexical meaning being dependent on the particular language.

## 8. Conclusions and Perspectives

In this paper, we described the role of preconceptual spectral and temporal cues in sound communication and in the emergence of meaning in speech and music, stressing the role of affective vocalizations as a common ancestral instrument in communicative interactions. In an attempt to search for shared components between speech and music, we have stressed their commonalities by defining speech and music as sensory rich stimuli. Their experience, moreover, involves different body channels, such as the face and the voice, but this bimodal approach has proven to be too restrictive. It has been argued, therefore, that an action-oriented approach is more likely to describe the reciprocity between multisensory processing and articulatory-motor routines as phonological primitives. As such, a distinction should be made between language and speech, with the latter being more centripetal in directing the attention of the listener to the sounding material itself, whereas language is mainly centrifugal in directing the attention away from the text to function referentially. There are, however, commonalities as well and the shared component between speech and music is not meaning, but sound. Therefore, to describe quite systematically the transition from sound to meaning in speech and music, one must stress the role of emotion and affect in early sound processing, the role of vocalizations and nonverbal affect burst in communicative sound comprehension, and the acoustic features of affective sound with a special emphasis on temporal and spectrographic cues as parts of speech prosody and musical expressiveness.

One of the major findings in this regard was a kind of hierarchy in the type of meaning that is conveyed, with a distinction between analog and digital usage of the sound. Especially, the role of affective prosody seems to be important here. As a typical example of analog processing, it goes beyond a mere discrete coding of speech and music, stressing the wider possibilities of sound-signal communications systems rather than relying merely on semantic content and propositional knowledge. As such, there seems to be a major ancestral function of affect burst, calls, protolanguage, and music which are related to several kinds of signaling, attention capturing, affective influence, and group cohesion. They hold a place in a developmental continuum at the phylogenetic and ontogenetic level. 

The view presented thus suggests that meaning in language and music is a complex phenomenon which is composed of hierarchically organized features, which are mostly related to the interpretation of acoustical cues by the nervous system. The bulk of this interpretation, moreover, is processed at an unconscious level. More studies are needed, however, to better understand the role of spectral and temporal cues as sources of information in the complex process of human communication. Inter-species and inter-cultural comparative studies are especially promising in this respect, but equally important are developmental investigations, which together with genetic research can elucidate the interconnection between the environmental and hereditary information in the process of the development of human vocal communication.
